# Effects of Auricular Acupressure on Glycemic Markers, Stress, and Sleep in Older Adult Patients With Type 2 Diabetes: A Randomized Controlled Trial

**DOI:** 10.1097/jnr.0000000000000683

**Published:** 2025-06-26

**Authors:** Hyejin LEE, Bomi KIM, Hyojung PARK

**Affiliations:** 1 College of Nursing, Ewha Womans University, Seoul, South Korea; 2 Department of Nursing, Chodang University, Muan, South Korea

**Keywords:** acupuncture therapy, diabetes mellitus, Type 2, sleep, stress

## Abstract

**Background:**

The prevalence of diabetes is a significant concern and is particularly impactful on the older adult population. Auricular acupressure is recognized as an effective complementary treatment for Type 2 diabetes.

**Purpose:**

The purpose of this study is to examine the effect of auricular acupressure on glycemic markers, stress, and sleep quality in older adults with Type 2 diabetes in South Korea.

**Methods:**

The study involved weekly acupressure therapy sessions for 8 weeks, with 25 participants in the intervention group and 26 in the placebo group. Specific acupoints associated with diabetes, sleep, and stress were targeted in the intervention group, while unrelated acupoints were used in the control group. Subjective indicators such as stress and sleep scales, along with objective measures such as blood tests, heart rate variability, and sleep activity recorders, were employed in the analysis.

**Results:**

Significant differences were observed in blood sugar (*F*=5.20, *p* <.001) and glycated hemoglobin (*Z*=−2.345, *p=*.019) between the two groups after administration of the acupressure therapy. However, no significant between-group differences were found in either glycated albumin or fructosamine. Also, activity in the sympathetic and parasympathetic nerves showed significant between-group variation. Although no significant between-group differences were found for subjective sleep indicators, notable changes in the number of awakenings, duration of awakening, REM sleep, and deep sleep conditions were identified.

**Conclusions:**

Although the effects are not strong, the findings suggests auricular acupressure influences glycemic index, stress, and sleep quality in older individuals with Type 2 diabetes positively. The results of this study support the potential of using auricular therapy as a nursing intervention in diabetes management.

## Introduction

Type 2 diabetes is characterized by decreased insulin secretion due to insulin resistance and the impaired secretion response of β cells, accounting for about 90% of diabetic patients (Ozougwu et al., 2013). Patients with Type 2 diabetes are susceptible to diabetic dyslipidemia and are at significantly elevated risk of vascular diseases (Arnold et al., 2020; Critchley et al., 2019; Kundu et al., 2017). In particular, the level of difficulty faced in controlling blood sugar levels increases with age, with older adults with diabetes at particularly high risk of experiencing reduced quality of life due to issues such as pressure ulcers, dementia, sarcopenia, immobility, and gait disorders as well as related complications (Yakaryilmaz & Öztürk, 2017; Zheng et al., 2018). As a result, blood sugar management to improve quality of life in older adults should be prioritized.

Stress affects the progression of Type 2 diabetes by activating the hypothalamic-pituitary-adrenal axis and the central sympathetic nervous system as well as by increasing cortisol secretion, which increases glycated hemoglobin levels and exacerbates short- and long-term complications (Erda et al., 2020; Kalra & Sharma, 2018). Moreover, diabetes affects the central nervous system, which can then influence neurobehavioral and neurotransmitter functions and induce sleep disturbances (Khandelwal et al., 2017; Reutrakul & Van Cauter, 2018). About two-thirds of patients with diabetes are affected by sleep disorders, which are known to cause negative emotions such as anxiety and depression that further reduce quality of life (Bopparaju & Surani, 2010; Hashimoto et al., 2020; Lee et al., 2017). Therefore, improving stress and sleep disorders is important in those with Type 2 diabetes.

Type 2 diabetes may be managed using pharmacological and nonpharmacological interventions, with nonpharmacological interventions particularly important for older adult patients in terms of minimizing the risks of hypoglycemia, hypotension, and drug interactions (Bopparaju & Surani, 2010). Auricular acupressure is a complementary and alternative approach that stimulates specific acupoints in the auricle to cause changes in the nervous and endocrine systems. Researchers have suggested that auricular acupressure may be effective in treating Type 2 diabetes (Oleson, 2013; Sun et al., 2021). In addition, auricular acupressure helps relieve stress and improve sleep quality by improving autonomic nervous system responses (Oleson, 2013). Acupressure is a simpler and less invasive intervention than acupuncture and has the advantage of being able to be performed independently by a nurse (Lee et al., 2010). Although the findings of prior studies have confirmed the effectiveness of auricular acupressure in improving Type 2 diabetes in older adults (Jang & Park, 2020; Liu et al., 2008; Wang et al., 2014), few studies have investigated the use of glycated hemoglobin, albumin, and fructosamine in glycemic control due to testing cost and inconvenience.

In light of the above, this study was designed to rigorously evaluate, using a placebo-controlled design, the effect of auricular acupressure on glycemic control, stress, and sleep in older patients with Type 2 diabetes.

## Methods

### Study Design

This single-blind, pretest-posttest, randomized controlled trial study was conducted after it was registered (KCT0008329) on March 31, 2023. The objective was to examine the effectiveness of auricular acupressure on older adult patients with Type 2 diabetes.

### Subjects

The participants were recruited from two lifelong education centers as well as churches and welfare centers located in Incheon City, Korea. After enrollment, the participants were randomly assigned to either the experimental or control group using a random number table generated by Random Allocation Software version 2.0 (Informer Technologies, Inc.).

The selection criteria were: (1) aged 65 or above; (2) diagnosed with Type 2 diabetes; (3) taking oral hypoglycemic medications or insulin; (4) sufficiently conscious to be able to fill out consent forms and questionnaires; and (5) scoring higher than 5 on the Pittsburgh Sleep Quality Index (PSQI). The exclusion criteria were: (1) currently taking sleeping pills; (2) diagnosed with a mental illness (depression, anxiety disorder); (3) having ear lesions or tape allergies; or (4) currently receiving other complementary or alternative therapies.

**Figure 1:**
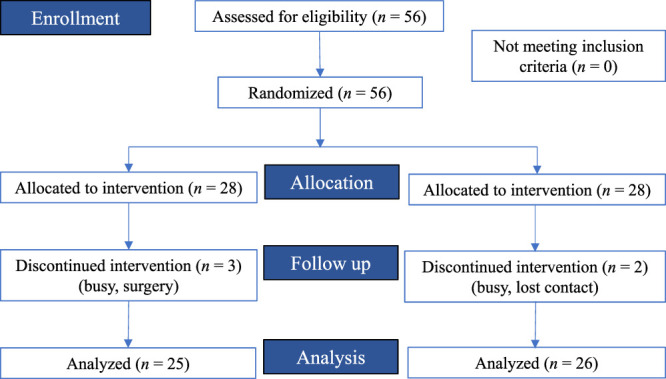
Consolidated Standards of Reporting Trials Flow Diagram for This Study

The minimum sample size for this study was calculated using G-power 3.1. In a previous study on heart rate variability (Montano et al., 1994), a minimum of 21 people were calculated for the experimental and control groups, respectively, when performing a *t* test and a one-tailed test with an effect size of .80, significance level of .05, and power of .80. After estimating a 25% maximum dropout rate, a sample of 56 people was recruited (28 in each group). Over the course of the study, in the experimental group, two people dropped out due to personal reasons and one due to scheduled surgery, while in the control group, one person dropped out due to personal reasons and one was eliminated due to loss of contact. Thus, the total number of participants was 51, with 25 in the experimental group and 26 in the control group (Figure 1).

### Intervention

In this study, a commercially available 1×1 cm patch containing a 1 mm black Melandryum firmum seed in the form of a small pellet was used. Melandryum firmum seeds, which are larger and harder than a mustard seed, are widely used to promote blood and meridian circulation. Before applying the patch, the condition of the ears of the participants was assessed, and a pad with 75% isopropyl alcohol was used to clean away substances such as oil and dead skin cells. After wiping the ears with an alcohol pad, patches containing one seed each were applied to the precise acupressure points. The participants pinched the patch areas with their thumb and index finger every two seconds until they felt a stinging sensation, after which they were told to pinch the patch similarly three times daily and once before bed, each time until they felt a slight tingle. Otherwise, the participants continued with their normal daily activities. They were asked to contact the researcher for reapplication in the event that one or more of the patches fell off.

**Figure 2:**
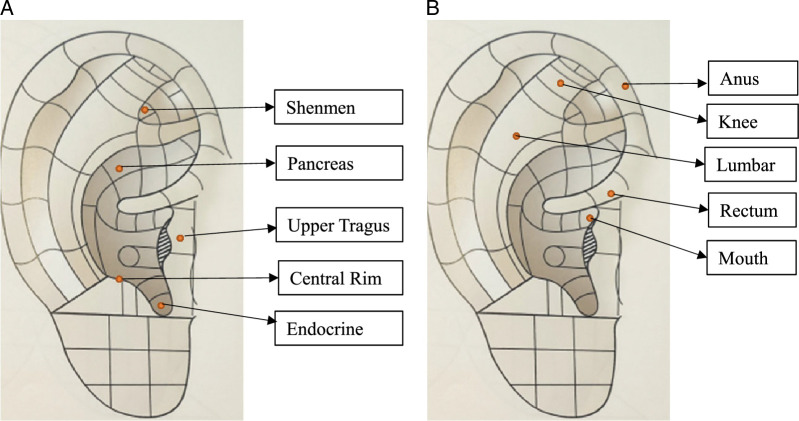
Auricular Acupoints Used in Experimental Group (A) and Control Group (B)

Each patch application session was 5 days, and the patches were applied alternately to the left and right ears each week. On the sixth day after each five-day patch application, the participants removed the patches on their own. For each application, discomfort or side effects, and skin integrity at previously pinched areas were examined. To reduce the dropout rate and increase willingness to participate, text messages were sent to the participants once per week to encourage consistent compliance with the study protocol. The procedure and time period were the same for the control group, with the exception that patches were applied to auricular reflex points. In this study, auricular acupressure was applied for 8 weeks to improve glycemic markers and stress levels. In several prior studies on the effects of auricular acupressure in improving glycemic markers, stress level, and sleep quality, the duration of application varied from 2 to 6 weeks, and most reported the intervention to be effective (Bi & Zheng, 2014; Liu et al., 2008; Qian et al, 2017). Based on the literature (Jang & Park, 2020; Lee et al., 2010; Wang et al., 2014) and prior research (Bi & Zheng, 2014; Liu et al., 2008; Qian et al., 2017) on auricular acupressure, five acupressure points each were selected in this study for the experimental and control groups in terms of their effectiveness on glycemic markers, stress, and sleep improvement. These included Shenmen, Pancreas, Endocrine, Upper tragus, and Central rim for the experimental group and acupressure points not related to stress and sleep improvement were selected such as Mouth, Rectum, Anus, Knee, and Lumbar for the control group. An auricular therapy specialist was consulted to confirm the accuracy of the acupressure points targeted. The targeted effect associated with each point is described as follows: Shenmen (sleeplessness, anxiety), Pancreas (diabetes, insomnia), Endocrine (endocrine diseases), Upper tragus (diabetic diseases), and Central rim (pituitary diseases). Placebo group used nonrelated points: Mouth (oral diseases), Rectum (digestive issues), Anus (anal diseases), Knee (joint disease), and Lumbar (back pain; Figure 2).

### Measurement

#### Glycemic markers

Glycemic markers included fasting glucose, glycated hemoglobin, albumin, and fructosamine. Fasting glucose was measured using a glucometer (Accu-Chek Guide, Roche, Germany). Glycated hemoglobin was used as an indicator of blood sugar control over the past 1–2 months, albumin as an indicator for the past 2–3 weeks, and fructosamine as an indicator for the past 1–2 weeks.

#### Perceived Stress Scale (PSS)

The PSS developed by Cohen et al. (1983) was used in this study to measure perceived stress. The PSS consists of 10 questions, each scored on a 5-point scale. The total possible scale score ranges from 0 to 40, with higher scores indicating higher perceived stress. Cohen et al. (1983) reported a Cronbach’s α for this scale of .84, with the Korean version translated by Park and Seo (2010) reporting a Cronbach’s α of .85.

#### Heart rate variability (HRV)

HRV was measured using uBioMacpa’s stress meter (uBioMacpa, Biosense Creative, Seoul, Korea) to identify stress index, sympathetic (low-frequency; LF), and parasympathetic (high-frequency; HF) activities as well as their balance (LF/HF ratio). Lower LF values (4.7–7.0 ms^2^) indicate more healthy sympathetic activity, higher HF values (3.5–6.8 ms^2^) indicate more healthy parasympathetic activity, and LF/HF ratios closer to 1.5 (0.8–1.5 ms^2^) indicate a more healthy autonomic balance.

#### Pittsburgh Sleep Quality Index (PSQI)

Sleep quality was assessed in this study using the PSQI-K adapted by Sohn et al., (2012) from the PSQI of Buysse et al. (1989) with Cronbach’s α values, respectively, of .84 and .83. The PSQI, a 19-item questionnaire, evaluates subjective sleep quality over the past month. Scores from seven categories are summed for a total scale score of 0–21 points, with higher scores indicating poorer sleep quality, and scores ≥5 suggestive of the presence of sleep issues.

#### Fitbit

Objective sleep quality and quantity were measured using a Fitbit tracker (Fitbit Charge HRTM, FitBit Inc., San Francisco, CA, USA) to accurately monitor sleep stages using heart rate and motion detection. Metrics recorded include total sleep time, awake time, light and deep sleep durations, and REM cycle over 1 day.

### Data Collection Procedure

Data collection occurred from March 21 to May 23, 2023. This study was implemented after approval from the institutional review board of Ewha Womans University (ewha-202301-0010-01). After describing the study purpose and procedures, the participants submitted written consent and underwent a pre-assessment. After the participants were instructed on how to answer the questionnaire, they filled out the questionnaires, providing information on their general characteristics, stress levels, and sleeping patterns. After completing the questionnaire, the institution where the participants applied for the study conducted a blood test. A clinical pathologist collected ~6 mL of blood under the guidance and supervision of a doctor. An institute specializing in the analysis of clinical trial specimens conducted all of the blood analysis work.

After the blood test, an additional 5-minute break was allowed to ensure accurate readings of heart rate variability. During the test, the participants were asked to breathe comfortably without speaking. During the pre-assessment, the researchers helped the participants affix a Fitbit tracker to their wrist and instructed them to either remove and return it themselves or to meet the researcher, who would remove it for them. All of the researchers in this study employed the same measurement methods and instruments to minimize measurement errors.

Blood glucose measurements were made weekly during the auricular acupressure treatment period, and a follow-up examination was performed during the last week after the conclusion of the 8-week auricular acupressure session. The posttest involved completing a questionnaire consisting of a stress scale and sleep quality scale, and a blood sample (6 mL) was collected by a clinical pathologist affiliated with an institution specializing in clinical trial sample analysis.

### Data Analysis

The collected data were analyzed using IBM SPSS Statistics 23.0 (IBM Corp., Armonk, NY, USA), with the statistical significance set to *p*<.05. The general characteristics of the study subjects were analyzed using real numbers and percentages, mean, and standard deviation. Experimental and control group characteristics were analyzed using chi-square and Fisher’s exact tests. The normal distribution of dependent variables was checked with Shapiro-Wilk using either an independent *t* test or Mann-Whitney *U* test. Time-based differences between groups were assessed using repeated measures analysis of variance, between-group changes were assessed using an independent *t* test and Mann-Whitney *U* test, and within-group changes were assessed using a paired *t* test and Wilcoxon test.

## Results

### Homogeneity Testing of General Characteristics

The mean ages in the intervention and control groups were, respectively, 73.00 (*SD*=8.41) and 70.62 (*SD*=4.66) years. No significant differences were observed between the two groups in terms of the following variables: age, diabetes duration, sex, educational background, economic status, exercise status, smoking, drinking, snacking, medication, insulin use, diabetes-related hospitalization, hypoglycemia within 1 month, diabetic complications, nondiabetic diseases, and use of complementary and alternative therapies (Table 1).

**Table 1:** Homogeneity of Variables Between Groups (N=51)

Variable	Mean±*SD*/ *n* (%)/Median (IQR)		*t/*χ^2^/*Z*	*p*
	Intervention (*n*=25)	Control (*n*=26)		
Age (year)	73.00±8.41	70.62±4.66	1.246	.221
Diabetes duration	10.30±8.87	8.64±6.34	0.773	.443
Sex				
Male	2 (8.0)	2 (7.7)		>.999 ^a^
Female	23 (92.0)	24 (92.3)		
Educational level				
Elementary	8 (32.0)	3 (11.5)		.184 ^a^
Middle school	4 (16.0)	10 (38.5)		
High school	10 (40.0)	10 (38.5)		
College or higher	3 (12.0)	3 (11.5)		
Economic status				
Upper	1 (4.0)	1 (3.8)		>.999 ^a^
Middle	16 (64.0)	17 (65.4)		
Lower	8 (32.0)	8 (30.8)		
Exercise			2.511	.113
No	11 (44.0)	6 (23.1)		
Yes	14 (56.0)	20 (76.9)		
Smoking			—	—
No	25 (100.0)	26 (100.0)		
Yes	0 (0.0)	0 (0.0)		
Alcohol consumption				.668 ^a^
No	23 (92.0)	22 (84.6)		
Yes	2 (8.0)	4 (15.4)		
Snacks				.419 ^a^
No	2 (8.0)	5 (19.2)		
Yes	23 (92.0)	21 (80.8)		
Oral diabetes medication use			0.899	.343
No	21 (84.0)	19 (73.1)		
Yes	4 (16.0)	7 (26.9)		
Insulin use				.610 ^a^
No	2 (8.0)	1 (3.8)		
Yes	23 (92.0)	25 (96.2)		
Diabetes-related hospitalization				>.999 ^a^
No	24 (96.0)	25 (96.2)		
Yes	1 (4.0)	1 (3.8)		
Low blood sugar within the previous month				>.999 ^a^
No	24 (96.0)	24 (92.3)		
Yes	1 (4.0)	2 (7.7)		
Diabetes complications				.110 ^a^
No	22 (88.0)	26 (100.0)		
Yes	3 (12.0)	0 (0.0)		
Concomitant diseases			2.946	.086
No	5 (20.0)	11 (42.3)		
Yes	20 (80.0)	15 (57.7)		
Experience using complementary or alternative therapies				
No	25 (100.0)	26 (100.0)	—	—
Yes	0 (0.0)	0 (0.0)		
Blood sugar test	124.24±30.46 115.00 (46.0)	125.56±24.15 119.00 (34.0)	−0.172	.865
HbA1c	6.78±0.61 6.60 (1.1)	6.69±0.66 6.55 (0.8)	−0.709	.478 ^b^
Albumin	16.62±3.40 15.20 (5.7)	16.42±2.77 15.55 (3.0)	−0.424	.672 ^b^
Fructosamine	293.56±43.47 280.00 (75.5)	310.81±45.17 302.50 (68.0)	−1.659	.097 ^b^
Perceived stress scale	22.80±6.85 21.00 (9.0)	24.27±6.40 25.00 (9.3)	−0.792	.432
LF	5.92±1.14 6.00 (1.4)	5.50±1.21 5.55 (1.8)	1.278	.207
HF	5.42±1.05 5.60 (1.8)	4.95±1.16 4.70 (1.8)	−1.698	.089 ^b^
LF/HF	1.10±0.17 1.10 (0.2)	1.06±0.28 1.10 (0.2)	−0.472	.637 ^b^
PSQI	7.68±3.04 7.00 (5.0)	8.12±3.46 8.00 (6.0)	−0.609	.543 ^b^
Total sleep time	430.36±136.11 441.00 (197.5)	471.58±122.24 457.00 (217.3)	−1.139	.260
Awake number	5.32±2.30 5.00 (2.5)	6.15±1.99 6.00 (2.3)	−1.345	.179 ^b^
Awake time	65.60±26.74 62.00 (43.5)	75.50±30.62 69.50 (53.3)	−1.204	.234
Rapid eye movement	79.84±31.98 82.00 (55.0)	70.04±34.51 79.00 (62.0)	0.969	.337
Low sleep	236.64±90.39 254.00 (105.5)	270.35±78.11 268.00 (88.5)	−1.219	.229
Deep sleep	48.28±27.44 39.00 (45.5)	54.58±19.99 50.00 (28.8)	−1.687	.092

*Note.* HF = parasympathetic activity; IQR = interquartile range; LF = sympathetic activity; LF/HF = parasympathetic and sympathetic balance activity; PSQI =Pittsburgh sleep quality index.

^a^ Fisher’s exact test; ^b^ Mann-Whitney *U* test.

In this study, the Shapiro-Wilk test for normality was performed to verify the homogeneity of the dependent variables between the groups. The Mann-Whitney *U* test was used to analyze the following: glycemic markers (blood sugar test; BST, HbA1c, albumin, and fructosamine) that did not satisfy the normality test; parasympathetic activity (HF) and autonomic balance (LF/HF), which are HRV markers; and sleep indices such as PSQI, AT, and DST. No statistically significant difference was detected in any of these variables (Table 1).

### Effect of Auricular Acupressure on the Glycemic Markers

The results obtained by analyzing the changes in blood glucose using repeated measurements are shown in Table 2. Blood glucose decreased significantly over time in both groups (*F*=5.20, *p*<.001). In the intervention group, the level of HbA1c decreased from 6.78±0.61 preintervention to 6.66±0.55 postintervention (*Z*=−2.390, *p=*.025), showing a statistically significant difference (*Z*=−3.974, *p*<.001). However, no statistically significant difference was detected in either albumin or fructosamine level between the two groups (Table 3).

**Table 2:** Blood Sugar Changes Over Time, by Group (N=51)

Blood Sugar Test	Mean±*SD*		Source	*F*	*P*
	Intervention	Control			
	(*n*=25)	(*n*=26)			
At baseline	124.24±30.46	125.56±24.15	Group	3.36	.073
After 1 wk	167.80±59.60	129.46±30.96			
After 2 wk	161.52±49.34	128.58±31.93			
After 3 wk	151.36±41.74	133.31±39.45	Time	7.18	<.001
After 4 wk	148.44±47.82	134.88±42.56			
After 5 wk	136.52±31.37	128.38±32.19			
After 6 wk	152.24±51.25	122.35±31.06			
After 7 wk	127.56±25.39	138.12±30.25	Group x Time	5.20	<.001
After 8 wk	114.28±23.66	118.77±23.53			

**Table 3:** Between-Group Differences in Blood Test Indicators, Stress, and Sleep Quality (N=51)

Variable/Group	Mean±*SD* Median (IQR)			Within Group		Between Group	
	Pretest	Posttest	Difference	*t* or *Z*	*p*	*t* or *Z*	*p*
HbA1c						−2.345	.019
Intervention	6.78±0.61 6.60 (1.1)	6.66±0.55 6.50 (0.9)	−0.12±0.24 −0.10 (0.3)	−2.390	.025		
Control	6.69±0.66 6.55 (0.8)	6.73±0.72 6.50 (0.8)	0.04±0.28 0.05 (0.3)	−3.974	<.001 ^a^		
Albumin						−1.359	.174
Intervention	16.62±3.40 15.20 (5.7)	17.50±3.25 16.60 (5.3)	0.88±1.71 1.50 (1.45)	−2.126	.033 ^a^		
Control	16.42±2.77 15.55 (3.0)	17.39±2.93 16.70 (2.7)	0.97±0.94 0.65 (0.9)	−0.202	.840 ^a^		
Fructosamine						−0.584	.559
Intervention	293.56±43.47 280.00 (75.5)	293.08±41.77 285.00 (46.0)	0.48±27.48 5.00 (46.0)	−0.229	.819 ^a^		
Control	310.81±45.17 302.50 (68.0)	309.50±44.23 302.00 (48.5)	−1.31±17.86 2.00 (22.0)	−0.485	.632		
PSS						−0.439	.663
Intervention	22.80±6.85 21.00 (9.0)	24.36±5.74 25.00 (6.0)	1.56±8.30 1.00 (12.5)	0.940	.357		
Control	24.27±6.40 25.00 (9.3)	25.04±6.13 26.50 (8.8)	0.77±3.55 0.00 (2.5)	1.106	.279		
LF						2.442	.018
Intervention	5.92±1.14 6.00 (1.4)	5.67±1.30 5.50 (1.9)	−0.25±1.18 −0.40 (1.35)	−1.086	.288		
Control	5.50±1.21 5.55 (1.8)	6.16±0.98 6.20 (1.3)	0.66±1.47 0.90 (1.7)	2.280	.031		
HF						−2.887	.004 ^b^
Intervention	5.42±1.05 5.60 (1.8)	5.13±1.03 4.90 (1.3)	−0.29±1.10 −0.30 (1.45)	−1.311	.202		
Control	4.95±1.16 4.70 (1.8)	5.62±1.04 5.35 (1.2)	0.67±1.48 0.70 (1.25)	−2.467	.014 ^a^		
LF/HF						−1.561	.119 ^b^
Intervention	1.10±0.17 1.10 (0.2)	1.16±0.14 1.15 (0.1)	0.06±0.25 0.10 (0.2)	−0.255	.081		
Control	1.06±0.28 1.10 (0.2)	1.09±0.18 1.10 (0.1)	0.03±0.18 0.00 (0.2)	−2.009	.045 ^a^		
PSQI						−1.200	.230 ^b^
Intervention	7.68±3.04 7.00 (5.0)	7.15±2.75 7.50 (4.0)	−0.53±2.65 −1.00 (4.5)	−3.306	.001 ^a^		
Control	8.12±3.46 8.00 (6.0)	7.15±2.75 7.50 (4.0)	−0.96±2.65 −1.00 (2.3)	−1.853	.076		
Total sleep time						0.327	.745
Intervention	430.36±136.11 441.00 (197.5)	414.80±105.83 413.00 (168.0)	−15.56±60.82 −13.00 (38.5)	−1.279	.213		
Control	471.58±122.24 457.00 (217.3)	462.89±109.77 474.50 (152.5)	−8.69±66.74 −13.50 (33.8)	0.664	.513		
Awake number						−2.860	.004 ^b^
Intervention	5.32±2.30 5.00 (2.5)	4.08±1.61 4.00 (2.0)	−1.24±1.05 −1.00 (1.5)	−3.830	<.001 ^a^		
Control	6.15±1.99 6.00 (2.3)	5.69±1.32 5.00 (1.3)	−0.46±0.86 −0.50 (1.0)	−2.524	.012 ^a^		
Awake time						3.510	.001
Intervention	65.60±26.74 62.00 (43.5)	55.88±24.65 49.00 (33.5)	−9.72±14.26 −11.00 (14.0)	−3.408	.002		
Control	75.50±30.62 69.50 (53.3)	76.77±25.11 72.00 (45.5)	1.27±9.34 1.00 (8.3)	0.693	.495		
REM						−2.167	.035
Intervention	79.84±31.98 82.00 (55.0)	86.32±24.94 87.00 (36.5)	6.48±10.63 7.00 (13.0)	3.048	.006		
Control	70.04±34.51 79.00 (62.0)	69.62±33.38 70.50 (57.3)	−0.42±13.78 0.50 (15.3)	−0.157	.877		
Low sleep						0.918	.363
Intervention	236.64±90.39 254.00 (105.5)	213.12±75.45 213.00 (104.5)	−23.52±53.53 −20.00 (30.0)	−2.197	.038		
Control	270.35±78.11 268.00 (88.5)	261.19±85.89 266.00 (93.5)	−9.15±57.72 −9.00 (19.3)	−0.809	.426		
Deep sleep						−3.397	.001 ^b^
Intervention	48.28±27.44 39.00 (45.5)	59.48±27.10 51.00 (41.5)	11.20±22.50 9.00 (11.0)	−3.147	.002 ^a^		
Control	54.58±19.99 50.00 (28.8)	54.15±15.15 51.00 (22.3)	−0.42±8.35 0.00 (6.0)	−0.504	.614 ^a^		

*Note.* HF = parasympathetic activity; IQR = interquartile range; LF = sympathetic activity; LF/HF = parasympathetic and sympathetic balance activity; PSS = perceived stress scale; PSQI = Pittsburgh sleep quality index; REM = rapid eye movement.

^a^ Wilcoxon rank-sum test; ^b^ Mann-Whitney *U* test.

### Effect of Auricular Acupressure on Stress Response

No significant between-group difference was detected in terms of PSS measured using the questionnaire (*t*=−.439, *p*=.663). On the other hand, according to the heart rate variability measurements, sympathetic nerve activity (LF) was 5.92±1.14 preintervention and 5.67±1.30 postintervention, revealing a statistically significant between-group difference in LF (*Z*=2.442, *p=*.018). Moreover, parasympathetic nerve activity (HF) decreased from 5.42±1.05 preintervention to 5.13±1.03 postintervention, revealing a statistically significant between-group difference in HF (*Z*=−2.887, *p=*.004). Notably, no significant between-group difference in autonomic balance (LF/HF) was detected (Table 3).

### Effect of Auricular Acupressure on Sleep Quality

In the experimental group, PSQI score decreased significantly from 7.68±3.04 preintervention to 7.15±2.75 after the eighth week of treatment (*Z*=−3.306, *p=*.001). Notably, the PSQI score in the control group also decreased significantly from 8.12±3.46 to 5.64±2.34 over the same period. Thus, no statistically significant between-group difference was found in sleep quality. Based on the measurements of different sleep stages acquired using a Fitbit tracker, significant between-group differences were found in terms of number of times awake (*Z*=−2.860, *p=*.004), AT (*t*=3.510, *p=*.001), REM time (*t*=−0.157, *p=*.877), and DST (*Z*=−3.397, *p=*.001). Notably, no significant between-group differences were observed for total sleep time (TST) or light sleep time (LST; Table 3).

## Discussion

This study was conducted to examine the glycemic markers (fasting blood glucose, glycated hemoglobin, fructosamine, and glycated albumin) in a sample of older adult patients with Type 2 diabetes and to determine the effect of auricular acupressure on both stress and quality of sleep. The results support auricular acupressure as effective in reducing blood sugar and stress levels and in improving sleep quality in this patient group. Fasting blood glucose and glycated hemoglobin were significantly lower in the experimental group than in the control group after 8 weeks of auricular acupressure treatment. Although it is difficult to make a direct comparison due to the lack of prior research into the auricular acupressure treatment of older adults with diabetes, this result is similar to that of a prior study in which 6 weeks of auricular acupressure treatment was shown to significantly reduce fasting blood glucose in older patients (Jang & Park, 2020) and that of a study reporting significantly reduced fasting blood glucose in a sample of patients aged 50 years and older after a 12-week auricular acupressure intervention (Bi & Zheng, 2014; Qian et al., 2017). Qian et al. applying an auricular acupressure intervention for 12 weeks, reported significant reductions in glycated hemoglobin levels similar to this study. However, another previous study (Jang & Park, 2020) using a 6-week auricular acupressure intervention reported no significant reduction in glycated hemoglobin levels. In a previous study (Qian et al., 2017) in which auricular acupressure was applied for 12 weeks, glycated hemoglobin in the experimental group was significantly reduced, showing a result similar to this study. Glycated hemoglobin is a marker that reflects the state of blood sugar control for 2–3 months; such a result can be interpreted as a difference in the study period. In this study, reductions in glycated albumin and fructosamine did not differ significantly between the two groups. As no prior research has considered either glycated albumin or fructosamine as glycemic markers, the results of this study cannot be compared to those of other studies. However, glycated albumin and fructosamine largely reflect the state of change during the first 1–2 weeks, as the indicator displaying blood sugar control state for 3 weeks and is influenced by short-term fluctuations in protein and glucose within plasma, resulting in differences with glycated hemoglobin (Freitas et al., 2017). The duration of this study was 8 weeks, and a significant difference was found in glycated hemoglobin results before and after the intervention, which showed a consistent decrease. No significant difference in blood glucose changes would be expected if the study were conducted for 2 weeks only. Therefore, another study should be conducted to determine the blood glucose changes over a 3-week period using glycated albumin and fructosamine as outcome indicators. Although the glycemic marker results differed between studies, auricular acupressure worked as an effective nursing intervention in terms of improving blood glucose levels when assessing fasting blood glucose and glycated hemoglobin. In this study, PSS was used as a subjective measure of stress level at the conclusion of the 8-week intervention. Based on the findings, no significant between-group difference was present in terms of questionnaire-assessed PSS score. PSS was previously confirmed to be a significant marker in one study (Lee & Park, 2021) that applied auricular acupressure for 8 weeks to alleviate stress in adults with hypertension and in another (Kim & Park, 2024) that assessed the level of stress in a sample of postmenopausal women. These studies thus disagree with this study, which found PSS not to be an effective subjective marker. This difference may be attributable to differences in disease type and participant ages among these studies, although the durations of the auricular acupressure intervention were all the same. In addition, subjective data markers are easy to collect and have high practicality, but their validity may be limited by the subjective judgments or situations of each respondent influencing their scoring decisions. In terms of more objective measures, heart rate variability (HRV) was used in this study as an objective measure of stress. The HRV is an indicator that shows phenomena related to the autonomic nervous system using sympathetic nervous system activity, parasympathetic nervous system activity, and autonomic nervous balance values taken during clinical visits (Montano et al., 1994). The HRV test highlighted a significant difference between the experimental group and control group in terms of sympathetic nervous system activity (LF) and parasympathetic nervous system activity (HF), which were similar to the findings of previous studies (Kim & Park, 2024; Lee & Park, 2021). Also, this study identified further differences when these findings were examined in detail. Regarding sympathetic nervous system activity, activity in the experimental group decreased, whereas that in the control group increased, confirming a lower level of stress in the experimental group. It was further confirmed that there was a significant difference in the parasympathetic activity, as activity in the experimental group decreased and that in the control group increased, supporting that stress levels had declined in the control group. These differences are attributable to the application of patches at the auricular acupressure points. The acupressure points of the control group used in the previous studies (Kim & Park, 2024; Lee & Park, 2021) as well as this study included helix 1–5, rectum, mouth, and tonsils, which are not related to pain, and these were different from the acupressure points used in this study. The acupressure points used for the control group of this study also included the lumbar spine and knee points. In older adults, lower back and knee joint pains are often chronic. Thus, the pain relief achieved using those acupressure points may have influenced the results. According to the principles of auricular acupressure, a problematic symptom is alleviated when the auricular reflex points of each disease are found and stimulated, reflecting the fact that the stimulus is transmitted to the brain and then to the body to relieve disease-related pain (Lee et al., 2010; Oleson, 2013). After stimulating certain auricular reflect points, comprehensive changes occur in the central nervous system, endocrine system, and peripheral nervous system. It seemed that stimulating acupressure points in these older patients caused changes in bodily systems that resulted in pain relief. PSQI was used in this study as a subjective marker to measure sleep quality after 8 weeks of auricular acupressure treatment. Although there was no significant between-group difference in PSQI, a significant difference in PSQI scores within the experimental group was found. These results are similar to a previous study that applied auricular acupressure to a group of institutionalized older adults for 6 weeks (Chang & Park, 2018). Moreover, both Kim et al. (2014) and Suen et al. (2019), who respectively applied 2- and 6-week auricular acupressure interventions on groups of older adults, reported significant between-group differences, confirming the effectiveness of auricular acupressure in improving sleep quality in older patients. With regard to the objective markers, a Fitbit tracker was used to measure TST, number of waking hours during sleep, AT, LST, DST, and REM cycle. In this study, significant between-group differences in the number of waking hours, AT, DST, and REM cycle were found. Most previous studies that examined the effect of auricular acupressure on older adults used subjective markers (self-report questionnaires) only, making it difficult to compare their results with this study. However, Suen et al. (2019), in which older adults with sleep disorders were investigated, reported reductions in AT, which somewhat agreed with the results of this study. Also, Lee and Park (2023), who investigated older adults with arthritis living in a nursing home, found between-group differences after the experimental group had received 8 weeks of auricular acupressure treatment, partially supporting the findings of this study. In addition, Lee and Park (2021), who investigated the effectiveness of applying auricular acupressure to homebound older adults for 6 weeks, reported an increase in DST and REM cycle in the experimental group, which was similar to the results of this study. However, LST significantly decreased as well, which was different from this study. In another previous study (Jang et al., 2019), in which auricular acupressure was applied for 8 weeks in a sample of older adult patients with degenerative knee arthritis, significant results were found in AT, LST, and DST but not for REM cycle, as the participants in that study frequently woke up during the night due to chronic pain, resulting in poor sleep efficiency (Rosa & Rustiaty, 2018). Therefore, the differences between the results of this study and prior studies may be largely attributable to differences in characteristics among the different samples, making comparisons of results difficult. As age increases, changes in sleep cycles lead to frequent awakenings at night and difficulties in returning to deep sleep (Yang & Kim, 2010). In the experimental group in this study, a significant decrease was found in the number of waking times during sleep, AT, and REM cycle, and an increase was found in DST, confirming the effect of auricular acupressure in reducing the number of awakenings and increasing DST in older individuals. Based on these results, this study confirmed that the auricular acupressure is effective in controlling sleep cycle changes and improving sleep quality in older adults with diabetes. However, in light of the differences in some of the findings between this and prior research, repeated studies on auricular acupressure should be conducted to clarify the effects of auricular acupressure on the health status, sleep patterns, and other changes in older adults.

Auricular acupressure, a noninvasive approach that continuously stimulates acupressure points using low-cost, readily available materials, is free of side effects such as pain, discomfort, and itching, making it safe and cost-effective for older individuals to use. Based on the findings of this study, applying auricular acupressure to enhance disease management, relieve stress, and improve sleep quality in older adults with Type 2 diabetes is recommended. Moreover, the use in this study of both subjective and objective data obtained from older individuals with Type 2 diabetes adds to the scientific support for the clinical use of auricular acupressure therapy. However, this study has some limitations. As the participants were recruited from lifelong education centers, churches, and welfare centers located in I City only, the results should be further validated on larger samples representing multiple regions in South Korea. Also, directly controlling factors such as individual lifestyle and habits known to influence diabetes, stress, and sleep was not possible in this study. In light of these limitations, the findings should be interpreted carefully.

### Conclusions

In this experimental study, the effects of auricular acupressure on glycemic markers, stress level, and sleep quality were investigated by implementing 8-week effective and placebo interventions, respectively, in two samples of older adults with Type 2 diabetes. The results support a positive effect of auricular acupressure on blood sugar management, stress level, and sleep quality. In the future, a study designed to examine the relative effect of auricular acupressure on different age groups (eg, college students, adults with Type 2 diabetes) should be conducted. Also, in future studies, it is recommended to recruit hospitalized patients or individuals in similarly controlled conditions, whose daily routines and surroundings are already consistently managed, to better control potential confounding variables such as lifestyle and environmental factors. Auricular acupressure may be incorporated into nursing interventions to manage diabetes, stress, and sleep quality in patients.

## References

[R1] Arnold S. V. Bhatt D. L. Barsness G. W. Beatty A. L. Deedwania P. C. Inzucchi S. E. Kosiborod M. Leiter L. A. Lipska K. J. Newman J. D. Welty F. K. , the American Heart Association Council on Lifestyle and Cardiometabolic Health and Council on Clinical Cardiology. (2020). Clinical management of stable coronary artery disease in patients with Type 2 diabetes mellitus: A scientific statement from the American Heart Association. Circulation, 141(19), e779–e806. https://doi.org/10.1161/CIR.0000000000000766. 32279539 10.1161/CIR.0000000000000766 PMC12204403

[R2] Bi Y. P. Zheng M. (2014). Clinical efficacy of auricular acupuncture as adjuvant therapy for Type 2 diabetes with deficiency of both qi and yin: A report of 50 cases in community. Journal of Anhui University Chinese Medicine, 33(2), 53–55.

[R3] Bopparaju S. Surani S. (2010). Sleep and diabetes. International Journal of Endocrinology, 2010, Article 759509. 10.1155/2010/759509 PMC2836131 20224753

[R4] Buysse D. J. Reynolds C. F. 3rd Monk T. H. Berman S. R. Kupfer D. J. (1989). The Pittsburgh Sleep Quality Index: A new instrument for psychiatric practice and research. Psychiatry Research, 28(2), 193–213. 10.1016/0165-1781(89)90047-4 2748771

[R5] Chang E. Park H. (2018). Effects of auricular acupressure therapy on musculoskeletal pain, depression, and sleep of the elderly in long-term care facilities. Journal of Korean Academy of Community Health Nursing, 29(2), 133–142. 10.12799/jkachn.2018.29.2.133 (Original work published in Korean)

[R6] Cohen S. Kamarck T. Mermelstein R. (1983). A global measure of perceived stress. Journal of Health and Social Behavior, 24, 385–396. 10.2307/2136404 6668417

[R7] Critchley J. A. Carey I. M. Harris T. DeWilde S. Cook D. G. (2019). Variability in glycated hemoglobin and risk of poor outcomes among people with Type 2 diabetes in a large primary care cohort study. Diabetes Care, 42(12), 2237–2246. 10.2337/dc19-0848 31582426

[R8] Erda R. Harefa C. M. Yulia R. Yunaspi D. (2020). The relationship of family support and stress with the quality of life of the elderly type II diabetes mellitus. Journal Keperawatan, 12(4), 1001–1010. 10.32583/keperawatan.v12i4.1034

[R9] Freitas P. A. C. Lethicia R. E. Joíza L. C. (2017). Glycated albumin: A potential biomarker in diabetes. Archives of Endocrinology and Metabolism, 61(3), 296–304. 10.1590/2359-3997000000272 28699985 PMC10118799

[R10] Hashimoto Y. Sakai R. Ikeda K. Fukui M. (2020). Association between sleep disorder and quality of life in patients with Type 2 diabetes: A cross-sectional study. BMC Endocrine Disorders, 20(1), Article No. 98. 10.1186/s12902-020-00579-4 PMC7325681 32605640

[R11] Jang M. Lim Y. M. Park H. (2019). Effects of auricular acupressure on joint pain, range of motion, and sleep in the elderly with knee osteoarthritis. Journal of Korean Academy of Community Health Nursing, 30(1), 79–89. 10.12799/jkachn.2019.30.1.79 (Original work published in Korean)

[R12] Jang M. Park H . (2020). The effects of auricular acupressure on postprandial glucose, HbA1c, blood lipids in aged patients with Type 2 diabetes mellitus. Journal of the Korea Academia-Industrial Cooperation Society, 21(8), 348–358. 10.5762/KAIS.2020.21.8.348 (Original work published in Korean)

[R13] Kalra S. Sharma S. K . (2018). Diabetes in the elderly. Diabetes Therapy, 9(2), 493–500. 10.1007/s13300-018-0380-x 29460258 PMC6104259

[R14] Khandelwal D. Dutta D. Chittawar S. Kalra S . (2017). Sleep disorders in Type 2 diabetes. Indian Journal of Endocrinology and Metabolism, 21(5), 758–761. 10.4103/ijem.IJEM_156_17 28989888 PMC5628550

[R15] Kim B. Park H . (2024). The effects of auricular acupressure on menopausal symptoms, stress, and sleep in postmenopausal middle-aged women: A randomized single-blind sham-controlled trial. Journal of Midwifery & Women’s Health, 69(1), 41–51. 10.1111/jmwh.13554 37549976

[R16] Kim J. Y. Ryu H. S. Nam S. H. Park K. S . (2014). Effects of auricular acupressure therapy on nocturia and insomnia in the elderly. The Korean Journal of Rehabilitation Nursing, 17(1), 1–9. 10.7587/kjrehn.2014.1 (Original work published in Korean)

[R17] Kundu D. Saikia M. Paul T. (2017). Study of the correlation between total lipid profile and glycosylated hemoglobin among the indigenous population of Guwahati. International Journal of Life Sciences Scientific Research, 3(4), 1175–1180.

[R18] Lee J. Kim S. Kim J. Park H . (2010). Auricular acupressure. Korean-Chinese Natural Healing Association. (Original work published in Korean)

[R19] Lee J. H. Park H . (2021). The effect of auricular acupressure on sleep in older adults with sleep disorders. Journal of Korean Gerontological Nursing, 23(2), 117–128. 10.17079/jkgn.2021.23.2.117 (Original work published in Korean)

[R20] Lee S. Park H. (2021). Effects of auricular acupressure on blood pressure and stress responses in adults with prehypertension. Journal of Korean Academy of Fundamental Nursing, 28(1), 174–185. 10.7739/jkafn.2021.28.2.174 (Original work published in Korean)

[R21] Lee S. W. H. Ng K. Y. Chin W. K. (2017). The impact of sleep amount and sleep quality on glycemic control in Type 2 diabetes: A systematic review and meta-analysis. Sleep Medicine Reviews, 31, 91–101. 10.1016/j.smrv.2016.02.001 26944909

[R22] Lee W. J. Park H. (2023). Effects of auricular acupressure on sleep and pain in elderly people who have osteoarthritis and live in nursing homes: A randomized, single-blind, placebo-controlled trial. Explore, 19(2), 214–222. 10.1016/j.explore.2022.07.001 35835645

[R23] Liu C. F. Yu L. F. Lin C. H. Lin S. C. (2008). Effect of auricular pellet acupressure on antioxidative systems in high-risk diabetes mellitus. The Journal of Alternative and Complementary Medicine, 14(3), 303–307. 10.1089/acm.2006.6064 18399759

[R24] Montano N. Ruscone T. G. Porta A. Lombardi F. Pagani M. Malliani A. (1994). Power spectrum analysis of heart rate variability to assess the changes in sympathovagal balance during graded orthostatic tilt. Circulation, 90(4), 1826–1831. 10.1161/01.cir.90.4.1826 7923668

[R25] Oleson T. (2013). Auriculotherapy manual: Chinese and Western systems of ear acupuncture. Elsevier Health Sciences.

[R26] Ozougwu J. C. Obimba K. C. Belonwu C. D. Unakalamba C. B. (2013). The pathogenesis and pathophysiology of Type 1 and Type 2 diabetes mellitus. Journal of Physiology and Pathophysiology, 4(4), 46–57. 10.5897/JPAP2013.0001

[R27] Park J. Seo Y. (2010). Validation of the perceived stress scale (PSS) on Samples of Korean university students. Korean Journal of Psychology: General, 29(3), 611–629. (Original work published in Korean)

[R28] Qian L. Lou R. Huang K. He L. (2017). Effect of combined auricular acupuncture therapy on blood glucose in Type 2 diabetics. Shanghai Journal of Acupuncture and Moxibustion, 36(5), 555–557. 10.13460/j.issn.1005-0957.2017.05.0555 (Original work published in Chinese)

[R29] Reutrakul S. Van Cauter E. (2018). Sleep influences on obesity, insulin resistance, and risk of Type 2 diabetes. Metabolism, 84, 56–66. 10.1016/j.metabol.2018.02.010 29510179

[R30] Rosa E. F. Rustiaty N. (2018). Affective disorders in the elderly: The risk of sleep disorders. International Journal of Public Health Science, 7(1), 33–38. 10.11591/ijphs.v7i1.9960

[R31] Sohn S. I. Kim D. H. Lee M. Y. Cho Y. W. (2012). The reliability and validity of the Korean version of the Pittsburgh Sleep Quality Index. Sleep and Breathing, 16, 803–812. 10.1007/s11325-011-0579-9 21901299

[R32] Suen L. K. Molassiotis A. Yueng S. K. W. Yeh C. H. (2019). Comparison of magnetic auriculotherapy, laser auriculotherapy, and their combination for treatment of insomnia in the elderly: A double-blinded randomised trial. Evidence-Based Complementary and Alternative Medicine. 2019(1), Article 3651268. 10.1155/2019/3651268 PMC6556291 31239857

[R33] Sun Y. Q. Peng S. Wang Y. L. Zhang S. Q. Wang W. R. (2021). The application progress of auricular therapy in diabetes. TMR Integrative Nursing, 5(3), 96–102. 10.12032/TMRIN2021062503

[R34] Wang S. Chen Z. Fu P. Zang L. Wang L. Zhai X. Gao F. Huang A. Zhang Y. (2014). Use of auricular acupressure to improve the quality of life in diabetic patients with chronic kidney diseases: A prospective randomized controlled trial. Evidence-Based Complementary and Alternative Medicine, 2014(1), Article 343608. 10.1155/2014/343608 PMC4276331 25574180

[R35] Yakaryilmaz F. D. Öztürk Z. A. (2017). Treatment of Type 2 diabetes mellitus in the elderly. World Journal of Diabetes, 8(6), 278–285. 10.4239/wjd.v8.i6.278 28694928 PMC5483426

[R36] Yang S. J. Kim J. S. (2010). Factors affecting the quality of sleep among community-dwelling elders. Journal of Korean Gerontological Nursing, 12(2), 108–118. (Original work published in Korean)

[R37] Zheng Y. Ley S. H. Hu F. B. (2018). Global aetiology and epidemiology of Type 2 diabetes mellitus and its complications. Nature Reviews Endocrinology, 14(2), 88–98. 10.1038/nrendo.2017.151 29219149

